# Unique aqueous self-assembly behavior of a thermoresponsive diblock copolymer†
†Electronic supplementary information (ESI) available: Materials and methods, polymer characterization techniques, SAXS models, supporting figures: GPC chromatograms, assigned ^1^H NMR spectra, calibration plot for determining the mean DP of the PDMAC precursor, temperature-dependent rheological studies, SIPLI rheology data, full spectra for the variable temperature ^1^H NMR spectroscopy study, apparent *z*-average diameter as a function of temperature as determined by DLS studies and apparent *z*-average diameter as a function of pH as determined by DLS studies. See DOI: 10.1039/c9sc04197d


**DOI:** 10.1039/c9sc04197d

**Published:** 2019-11-12

**Authors:** Sarah J. Byard, Cate T. O'Brien, Matthew J. Derry, Mark Williams, Oleksandr O. Mykhaylyk, Adam Blanazs, Steven P. Armes

**Affiliations:** a Department of Chemistry , University of Sheffield , Dainton Building , Brook Hill , Sheffield , South Yorkshire S3 7HF , UK . Email: s.p.armes@sheffield.ac.uk; b BASF SE , GMV/P-B001 , 67056 Ludwigshafen , Germany

## Abstract

It is well-recognized that block copolymer self-assembly in solution typically produces spheres, worms or vesicles, with the relative volume fraction of each block dictating the copolymer morphology. Stimulus-responsive diblock copolymers that can undergo either sphere/worm or vesicle/worm transitions are also well-documented. Herein we report a new amphiphilic diblock copolymer that can form spheres, worms, vesicles or lamellae in aqueous solution. Such self-assembly behavior is unprecedented for a single diblock copolymer of fixed composition yet is achieved simply by raising the solution temperature from 1 °C (spheres) to 25 °C (worms) to 50 °C (vesicles) to 70 °C (lamellae). Heating increases the degree of hydration (and hence the effective volume fraction) of the core-forming block, with this parameter being solely responsible for driving the sphere-to-worm, worm-to-vesicle and vesicle-to-lamellae transitions. The first two transitions exhibit excellent reversibility but the vesicle-to-lamellae transition exhibits hysteresis on cooling. This new thermoresponsive diblock copolymer provides a useful model for studying such morphological transitions and is likely to be of significant interest for theoretical studies.

## Abstract 2

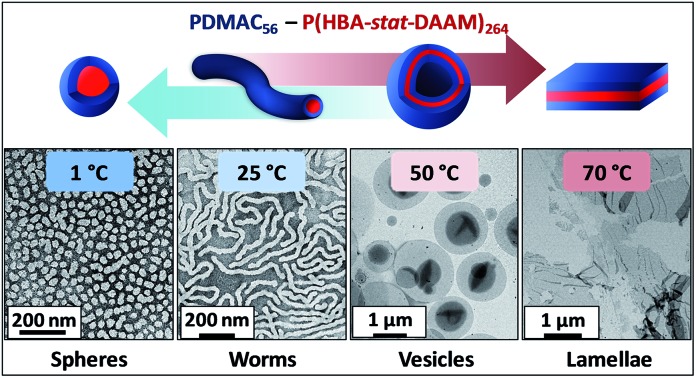
A new amphiphilic diblock copolymer prepared *via* polymerization-induced self-assembly forms spheres, worms, vesicles or lamellae in aqueous solution on adjusting the temperature.

## Introduction

Block copolymer self-assembly has been extensively studied because it offers a wide range of potential applications, including thermoplastic elastomers,^1^ catalysts,^2^ toughening agents for epoxy resins,^3^ ultrafiltration membranes,^4^ lithographic materials,^5^ high-density arrays^6^,^7^ and drug delivery.^8^–^10^ It is well-known that the self-assembly of diblock copolymers in the solid state can result in a wide range of morphologies.^7^,^11^–^15^ Similarly, diblock copolymers can self-assemble in solution when the solvent is selective for one of the two blocks. In this case, the final morphology depends on the relative volume fractions of each block,^16^ the selectivity of the solvent(s) for each block,^17^ and the copolymer concentration.^18^,^19^ It is well-documented that the three main copolymer morphologies formed in solution are spheres,^16^ worms^19^,^20^ and vesicles^21^ but other morphologies such as rods,^22^–^24^ toroids^25^ and lamellae^26^ have also been reported.

Traditionally, block copolymer self-assembly in solution has been achieved *via* various post-polymerization processing strategies.^16^,^20^–^23^ However, over the past decade increasing attention has been paid to polymerization-induced self-assembly (PISA).^19^,^24^,^27^–^30^ Most PISA syntheses reported in the literature are based on reversible addition–fragmentation chain transfer (RAFT) polymerization.^29^,^31^–^44^ The versatility of this radical-based chemistry has enabled the convenient preparation of a wide range of well-defined functional block copolymers in the form of concentrated dispersions.^27^–^29^ More specifically, PISA syntheses based on RAFT dispersion polymerization has enabled the synthesis of diblock copolymer nano-objects that can exhibit thermoresponsive behavior.^19^,^45^–^50^ For example, cooling an aqueous amphiphilic diblock copolymer worm gel below ambient temperature induces a worm-to-sphere transition (and concomitant degelation).^19^ Alternatively, heating the analogous hydrophobic copolymer worms in non-aqueous media can induce the same change in copolymer morphology.^48^ In each case, this change in morphology can be rationalized in terms of surface plasticization of the nano-objects by solvent molecules, which leads to a subtle change in the fractional packing parameter for the copolymer chains.^51^–^53^ There have been a couple of reports of diblock copolymers that can form more than one morphology in a given solvent^18^ or a binary mixture of solvents under certain conditions.^17^ However, as far as we are aware, there have been no reports of a *single* (*i.e.* fixed composition) diblock copolymer in any solvent that is capable of crossing *three* phase boundaries to form spheres, worms, vesicles or lamellae.

**Fig. 1:**
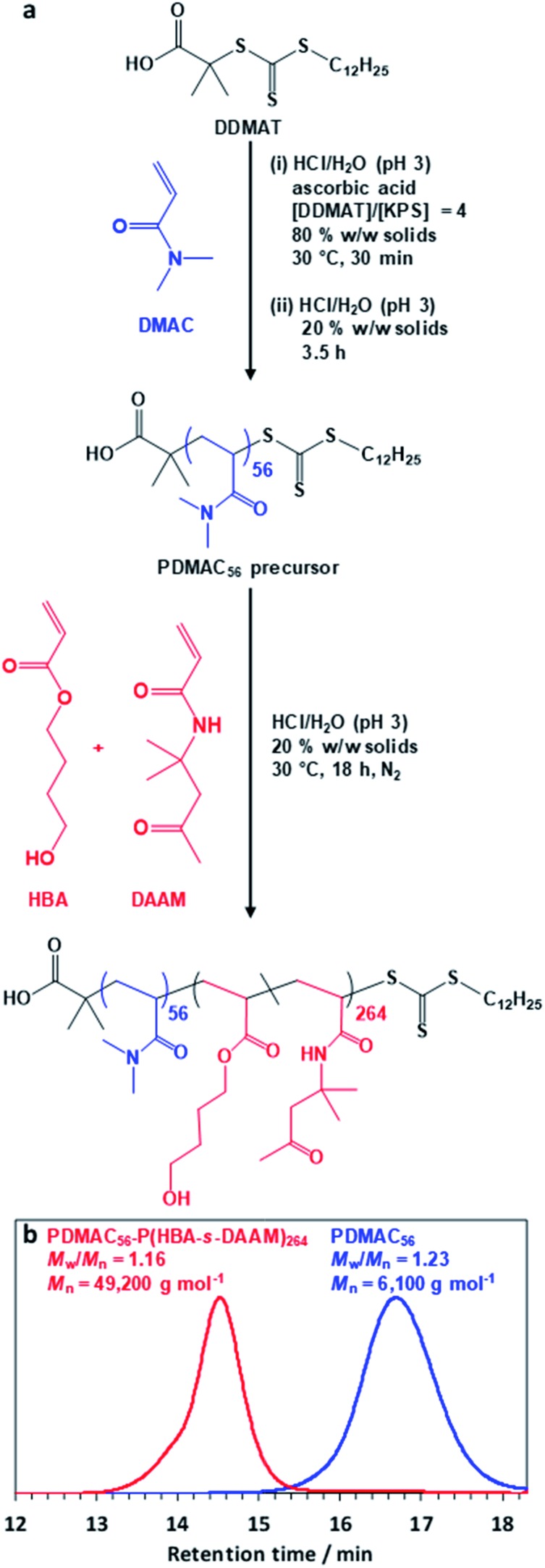
(a) Block copolymer synthesis *via* polymerization-induced self-assembly (PISA). Reaction scheme for the synthesis of the PDMAC_56_ precursor *via* RAFT solution polymerization of DMAC at 30 °C using a DDMAT chain transfer agent and a redox initiator [potassium persulfate (KPS) plus ascorbic acid]. Subsequent PDMAC_56_ chain extension with a binary mixture of HBA (80 mol%) and DAAM (20 mol%) *via* RAFT aqueous dispersion copolymerization at pH 3 produced a well-defined PDMAC_56_–P(HBA-*stat*-DAAM)_264_ diblock copolymer. (b) DMF GPC data obtained for the PDMAC_56_ precursor and final PDMAC_56_–P(HBA-*stat*-DAAM)_264_ diblock copolymer (refractive index detector; expressed relative to a series of poly(methyl methacrylate) calibration standards).

Such rich phase behavior is demonstrated herein for a new poly(*N*,*N*-dimethylacrylamide)-*block*-poly(4-hydroxybutyl acrylate-*stat*-diacetone acrylamide) [PDMAC–P(HBA-*stat*-DAAM)] amphiphilic diblock copolymer that is prepared directly in water under mild conditions (see Fig. 1). The sphere/worm and worm/vesicle phase transitions can be achieved by simply varying the temperature of the resulting aqueous copolymer dispersion and proceed both rapidly and reversibly even when conducted at copolymer concentrations as low as 0.10% w/w. Moreover, a vesicle-to-lamellae transition is also observed for this system, although this latter thermal transition is characterized by significant hysteresis during the cooling cycle.

## Results and discussion

The diblock copolymer used in this study was prepared *via* PISA using a highly convenient one-pot RAFT aqueous dispersion polymerization formulation (Fig. 1a). First, *N*,*N′*-dimethylacrylamide (DMAC) was polymerized in an 80% w/w aqueous solution at 30 °C using a trithiocarbonate-based RAFT agent combined with a low-temperature redox initiator. The initial highly concentrated aqueous solution was required to ensure solubility of the RAFT agent. After 30 min, the reaction mixture was diluted to 20% w/w in order to lower its solution viscosity. After 4 h, a small aliquot of the resulting water-soluble PDMAC precursor was removed for analysis. Gel permeation chromatography (GPC; DMF eluent, 60 °C) analysis indicated a relatively narrow molecular weight distribution (*M*_w_/*M*_n_ = 1.23, see Fig. 1b) while ^1^H NMR spectroscopy studies confirmed that more than 99% DMAC conversion had been achieved (Fig. S1, ESI†). The mean degree of polymerization (DP) of this PDMAC precursor was determined to be 56 by end-group analysis using UV spectroscopy (see Fig. S2, ESI†).

This PDMAC_56_ precursor was then chain-extended *in situ* by statistical copolymerization of a mixture of 4-hydroxybutyl acrylate (HBA; 80 mol%) and diacetone acrylamide (DAAM; 20 mol%) at 20% w/w solids to produce a PDMAC_56_–P(HBA-*stat*-DAAM)_264_ diblock copolymer, where the subscripts refer to the mean DPs of each block. GPC analysis indicated efficient chain extension and a relatively narrow molecular weight distribution (*M*_w_/*M*_n_ < 1.20) for the final diblock copolymer (see Fig. 1b). ^1^H NMR spectroscopy studies confirmed essentially full conversion for the HBA and DAAM comonomers (>99%; see Fig. S1, ESI†) and also that the expected composition for the core-forming P(HBA-*stat*-DAAM)_264_ block (78 ± 2 mol% HBA) was obtained within experimental error.

**Fig. 2:**
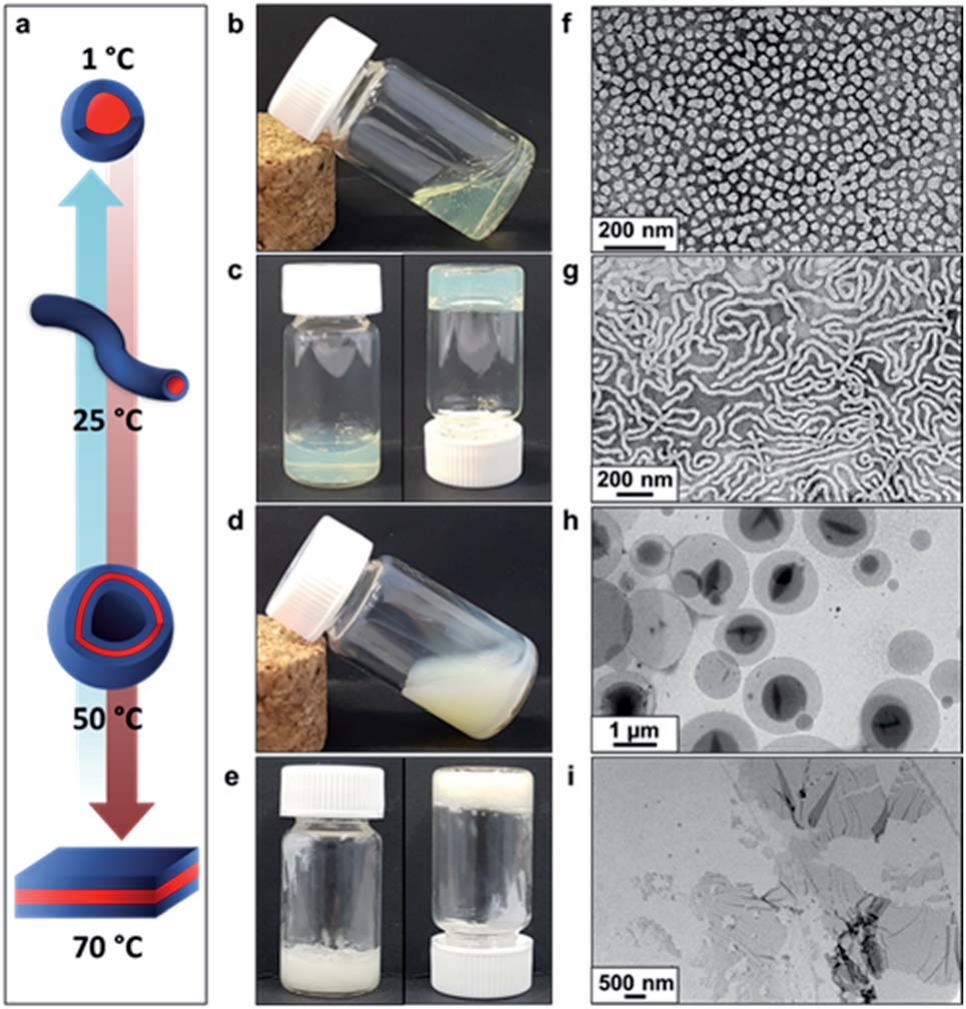
Thermally-induced diblock copolymer morphology transitions in aqueous solution. (a) Schematic representation of the reversible morphological transitions that occur for a 20% w/w aqueous dispersion of PDMAC_56_–P(HBA-*stat*-DAAM)_264_ diblock copolymer nano-objects on varying the temperature from 1° to 70 °C. Digital images show the physical appearance of this aqueous dispersion: (b) on cooling to 1 °C for 30 min, (c) at ambient temperature (25 °C), (d) on heating to 50 °C for 30 min and (e) on heating to 70 °C for 30 min. TEM images recorded for 0.10% w/w aqueous dispersions of PDMAC_56_–P(HBA-*stat*-DAAM)_264_ using uranyl formate as a negative stain after covalent stabilization at the desired temperature using adipic acid dihydrazide (ADH) at a DAAM/ADH molar ratio of 1.0: (f) spheres (crosslinked at 1 °C), (g) worms (crosslinked at 25 °C), (h) vesicles (crosslinked at 50 °C) and (i) lamellae (crosslinked at 70 °C).

Recent *in silico* studies and preliminary experimental data suggest that HBA should be a suitable core-forming block for aqueous PISA.^54^ Indeed, the 20% w/w dispersion of PDMAC_56_–P(HBA-*stat*-DAAM)_264_ diblock copolymer nano-objects prepared herein formed a free-standing gel at 25 °C (Fig. 2). On cooling to 1 °C, degelation occurred to produce a transparent free-flowing dispersion. On heating to 50 °C, a turbid, free-flowing dispersion was formed, while a turbid paste was formed at 70 °C. To examine the copolymer morphologies associated with these thermal transitions, transmission electron microscopy (TEM) studies were performed on the PDMAC_56_–P(HBA-*stat*-DAAM)_264_ diblock copolymer nano-objects. Crosslinking of the P(HBA-*stat*-DAAM)_264_ block was conducted at the desired temperature using adipic acid dihydrazide (ADH) at pH 3, as previously reported for PDMAC–PDAAM diblock copolymer nano-objects.^55^ Such covalent stabilization was essential to obtain high-quality images: in the absence of any cross-linking, the relatively low glass transition temperature of the core-forming block simply led to film formation on the TEM grid. Moreover, crosslinking also eliminated the thermoresponsive behavior of this diblock copolymer, hence preserving the copolymer morphology at any desired temperature. Thus this cross-linking protocol enabled visualization of pure spheres, worms, vesicles or lamellae after covalent stabilization at 1, 25, 50 or 70 °C, respectively (Fig. 2f–i).

**Fig. 3:**
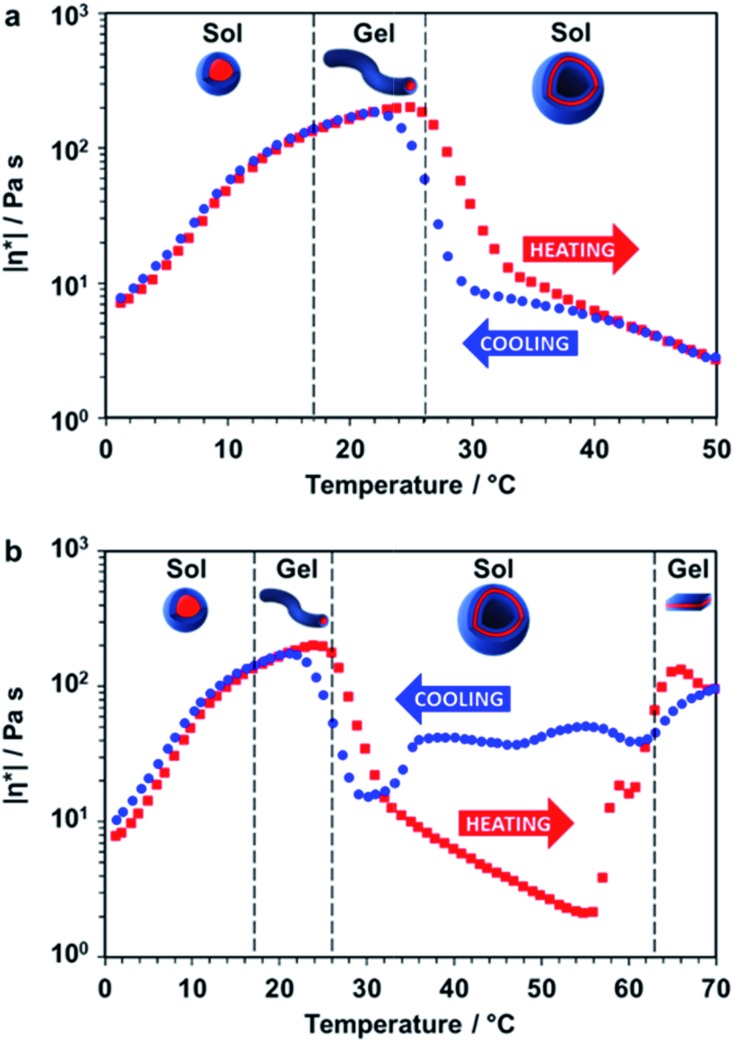
Thermally-induced change in complex viscosity for an aqueous dispersion of linear diblock copolymer nano-objects. Temperature-dependent rheological studies for a 20% w/w aqueous dispersion of PDMAC_56_–P(HBA-*stat*-DAAM)_264_ nano-objects at an applied strain of 1.0% and an angular frequency of 1.0 rad s^–1^. The dispersion was equilibrated at 1 °C for 15 min prior to heating. The black dashed lines indicate the sol–gel transitions observed on heating as determined from the *G*′ and *G*′′ values (Fig. S3, ESI†). (a) Complex viscosity (|η*|) *vs.* temperature data obtained for a thermal cycle from 1 °C to 50 °C to 1 °C at a heating/cooling rate of 1 °C min^–1^ [■ heating curve, [black circle] cooling curve]. (b) Complex viscosity *vs.* temperature data for a thermal cycle over an expanded temperature range of 1 °C to 70 °C to 1 °C at a heating/cooling rate of 1 °C min^–1^ [■ heating curve, [black circle] cooling curve].

Rheology studies were conducted on a 20% w/w aqueous dispersion of the *linear* (*i.e.* non-crosslinked) PDMAC_56_–P(HBA-*stat*-DAAM)_264_ nano-objects as a function of temperature (Fig. 3 and S3, ESI†). As expected, a low-viscosity fluid was formed at 1 °C owing to the presence of free-flowing spherical nano-objects. Warming to ambient temperature induced a sol–gel transition, producing a soft, transparent free-standing gel. This indicates the formation of highly anisotropic worms, with multiple inter-particle contacts producing a 3D network.^56^ The storage modulus (*G*′) exceeds the loss modulus (*G*′′) at 17 °C, which corresponds to the critical gelation temperature (CGT) (Fig. S3, ESI,† for *G*′ and *G*′′ data). A maximum gel viscosity was observed at 25 °C. Further heating led to a significant *reduction* in viscosity (and a concomitant increase in turbidity) owing to the formation of vesicles. These sphere-to-worm and worm-to-vesicle transitions proved to be remarkably reversible, with relatively little hysteresis being observed at heating/cooling rates of 1 °C min^–1^ (Fig. 3a). Heating the turbid, free-flowing vesicular dispersion above 50 °C initially caused a further reduction in the complex viscosity (see Fig. 3b). However, the dispersion became a turbid paste between 63 °C and 70 °C and the complex viscosity increased by approximately two orders of magnitude, which corresponds to the formation of lamellae (see Fig. 2i). Significant hysteresis was observed for the lamellae-to-vesicle transition on cooling, but good reversibility was observed below approximately 22 °C.

Further evidence for these four types of diblock copolymer nano-objects was obtained using shear-induced polarized light imaging (SIPLI), see Fig. S4.† This opto-rheological technique has been recently used to demonstrate the alignment of *anisotropic* nano-objects such as block copolymer worms and lamellae at a certain critical rate of applied shear.^57^ Thus, the SIPLI image recorded under continuous shear flow at 3 °C appears dark (see Fig. S4†); this is consistent with the presence of isotropic spheres, which cannot be aligned. A characteristic Maltese cross is observed under this shear flow at approximately 20 °C, indicating the formation (and shear-induced alignment) of highly anisotropic worms. This distinctive pattern disappears at 33 °C owing to the formation of isotropic vesicles while a new Maltese cross (with associated multiple colors arising from strong birefringence) is obtained at 45 °C. The latter feature indicates the presence of anisotropic lamellae that have either a *perpendicular* (with the lamellar normal parallel to the neutral direction of the flow) or *transverse* (with the lamellar normal parallel to the velocity direction of the flow) orientation.^58^ [N.B. The above characteristic temperatures required to induce formation of block copolymer worms, vesicles and lamellae do not match those indicated by the oscillatory rheology data shown in Fig. 3. This is because the applied (continuous) shear is significantly greater in the latter case, which promotes the formation of worms, vesicles and lamellae under milder conditions, *i.e.* lower temperatures].

**Fig. 4:**
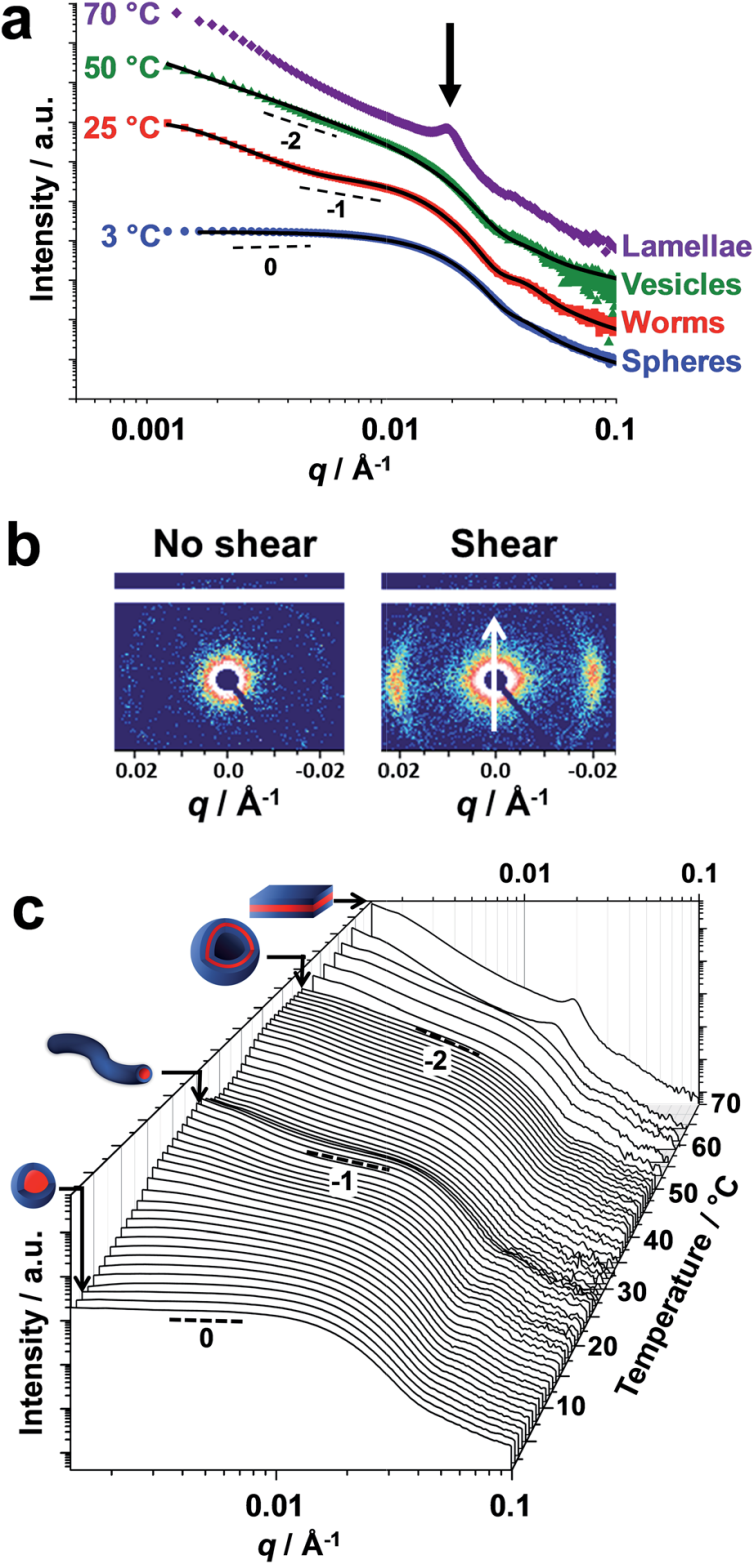
Small-angle X-ray scattering studies of linear diblock copolymer nano-objects. (a) Double-logarithmic plot of SAXS patterns recorded for a 1.0% w/w aqueous dispersion of thermoresponsive PDMAC_56_–P(HBA-*stat*-DAAM)_264_ nano-objects at 3 °C (blue data), 25 °C (red data), 50 °C (green data) and 70 °C (purple data). The black lines indicate the data fits obtained using appropriate scattering models. For guidance, black dashed lines indicate zero, –1 and –2 gradients, while the blue, green and purple data sets are offset by arbitrary factors to aid clarity. (b) 2D SAXS patterns recorded either at zero shear or under applied shear (direction indicated by the white arrow) during rheo-SAXS experiments conducted on a 20% w/w aqueous dispersion of PDMAC_56_–P(HBA-*stat*-DAAM)_264_ nano-objects at 63 °C. (c) SAXS patterns recorded for a 1.0% w/w aqueous dispersion of thermoresponsive PDMAC_56_–P(HBA-*stat*-DAAM)_264_ nano-objects between 1 and 70 °C using a heating rate of 1 °C min^–1^. For guidance, black dashed lines indicate zero, –1 and –2 gradients.

Small-angle X-ray scattering (SAXS) studies were conducted on a 1.0% w/w aqueous dispersion of linear PDMAC_56_–P(HBA-*stat*-DAAM)_264_ nano-objects as a function of temperature at pH 3 (Fig. 4). The gradient in the low *q* region of an *I*(*q*) *vs. q* plot (where *I*(*q*) is the scattering intensity and *q* is the scattering vector) is characteristic of the predominant copolymer morphology.^59^ This gradient is close to zero at 1 °C, which suggests the presence of spheres. At 25 °C, the gradient shifts towards –1, indicating the formation of highly anisotropic worms.^19^,^48^ On raising the temperature to 50 °C, the low *q* gradient increases to –2, which is characteristic of bilayer (or vesicle) formation. At 70 °C, the diffraction peak observed at *q* = 0.019 Å^–1^ suggests stacked lamellae sheets (see Fig. 4a, black arrow). Analysis of the SAXS data shown in Fig. 4a indicated a core diameter of 22.8 nm for the spheres, which is consistent with an overall hydrodynamic DLS diameter of 33 nm recorded at the same temperature. A core cross-section diameter of 23.0 nm was determined for the worms; this is consistent with TEM studies (26 ± 3 nm) if the highly deformable nature of the core-forming block is taken into consideration. The mean vesicle membrane thickness was 17.2 nm, which indicates significant inter-digitation of the structure-directing hydrophobic P(HBA-*stat*-DAAM)_264_ chains.^60^

**Fig. 5:**
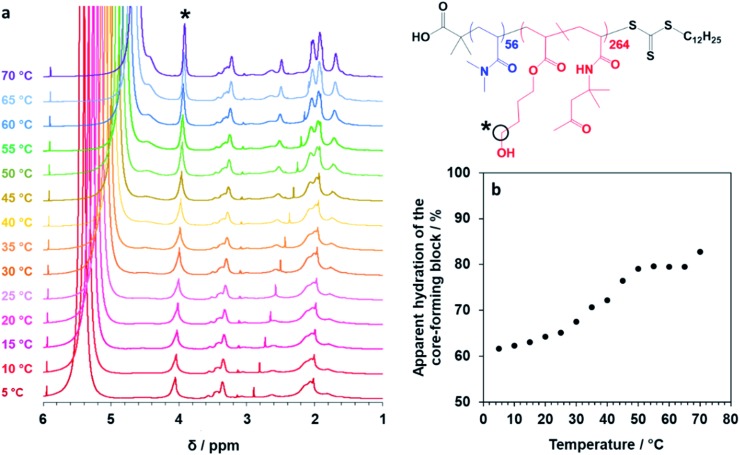
Variable temperature NMR studies of linear thermoresponsive diblock copolymer nano-objects. (a) Normalized ^1^H NMR spectra recorded for a 20% w/w aqueous dispersion of PDMAC_56_–P(HBA-*stat*-DAAM)_264_ nano-objects on heating from 5 °C to 70 °C. For clarity, only partial spectra in the 1–6 ppm range are shown (see Fig. S5, ESI,† for the full spectra). The signal marked with an asterisk is assigned to the two CH[combining low line]_2_–OH protons on the HBA residues: the gradual increase in its intensity on heating indicates progressively greater hydration of the core-forming block at higher temperature. (b) Apparent degree of hydration of the hydrophobic P(HBA-*stat*-DAAM)_264_ block as a function of temperature (with 100% hydration corresponding to the true composition of this structure-directing block, as calculated by ^1^H NMR spectroscopy studies of the molecularly-dissolved copolymer chains in CD_3_OD).

Finally, the mean distance between the stacked lamellae sheets was determined to be 33 nm as calculated from the diffraction peak observed at *q* = 0.019 Å^–1^. Fig. 4b shows 2D SAXS patterns recorded under applied shear and also at zero shear during rheo-SAXS experiments conducted at 63 °C (see ESI† for further details). The distinctly anisotropic pattern obtained under applied shear clearly indicates the presence of lamellae in a perpendicular orientation.^58^Fig. 4c shows a series of SAXS patterns recorded for a 1.0% w/w aqueous dispersion of PDMAC_56_–P(HBA-*stat*-DAAM)_264_ nano-objects on heating from 1 °C to 70 °C at a heating rate of 1 °C min^–1^. Clearly, there is a gradual increase in the low *q* gradient as the initial spheres are converted into first worms and then vesicles. The diffraction peak assigned to stacked lamellae is observed at 64 °C and 70 °C (Fig. 4c). This data set confirms that the interconversion between these four copolymer morphologies occurs rapidly on relatively short time scales, even at rather low copolymer concentration. In summary, the TEM images (Fig. 2), oscillatory rheology studies (Fig. 3), SIPLI measurements (Fig. S4†) and SAXS experiments (Fig. 4) provide compelling evidence for inter-conversion between spheres, worms, vesicles and lamellae for this PDMAC_56_–P(HBA-*stat*-DAAM)_264_ diblock copolymer. Variable temperature ^1^H NMR spectroscopy studies were conducted to examine the mechanism for this unique thermoresponsive behavior. Accordingly, a 20% w/w aqueous copolymer dispersion was heated from 5 °C to 70 °C with spectra being recorded at 5 °C intervals and normalized relative to an external standard (pyridine). Partial ^1^H NMR spectra are shown in Fig. 5 (see Fig. S5, ESI,† for the full spectra). ^1^H NMR signals assigned to the core-forming P(HBA-*stat*-DAAM)_264_ chains become more prominent at higher temperature, indicating progressively greater hydration for this weakly hydrophobic structure-directing block (Fig. 5a). Such spectral changes can be quantified by normalizing the integrated intensity of the two CH[combining low line]_2_–OH protons assigned to the HBA repeat units relative to that of the external standard. This approach enables the *apparent* degree of hydration of the HBA repeat units within the P(HBA-*stat*-DAAM)_264_ core-forming block to be calculated at any given temperature. This parameter is expressed as a percentage of the maximum value determined by ^1^H NMR spectroscopy using CD_3_OD (the PDMAC_56_–P(HBA-*stat*-DAAM)_264_ chains are molecularly dissolved in this solvent); it increases from 62% to 83% on heating a 20% w/w aqueous copolymer dispersion from 5 °C to 70 °C (Fig. 5b).

Clearly, the P(HBA-*stat*-DAAM)_264_ block is partially hydrated at all temperatures and its degree of hydration *increases* at higher temperature. This is attributed to the temperature dependence of the Flory–Huggins *χ* parameter for the interaction between the P(HBA-*stat*-DAAM)_264_ chains and water. As this hydrophobic block becomes progressively more hydrated, its volume fraction increases relative to that of the hydrophilic PDMAC stabilizer block. This leads to a subtle increase in the fractional packing parameter, *P*, for the copolymer chains,^51^,^52^ which accounts for the evolution in morphology from spheres to worms to vesicles to lamellae that is observed on heating. These morphological transitions are ultimately reversible on cooling, although significant hysteresis is observed for the initial lamellae-to-vesicle transformation (see Fig. 3b). Interestingly, similar thermoresponsive behavior has been recently predicted for a single diblock copolymer composition by Borisov and co-workers.^61^

Dynamic light scattering (DLS) was used to determine the sphere-equivalent diameter for a 0.10% w/w PDMAC_56_–P(HBA-*stat*-DAAM)_264_ aqueous dispersion during a thermal cycle from 2 °C to 50 °C to 2 °C (Fig. S6, ESI†). These DLS data suggest that both the sphere-to-worm and worm-to-vesicle thermal transitions occur rapidly and reversibly, with minimal hysteresis being observed even at copolymer concentrations as low as 0.10% w/w. The excellent reversibility observed under such conditions is attributed to the relatively high mobility of the acrylic-rich core-forming block, which has a relatively low glass transition temperature.

Finally, raising the solution pH from pH 3 to pH 7 at 20 °C causes a reversible reduction in the sphere-equivalent particle diameter from 333 nm (worms) to 38 nm (spheres) as judged by DLS (see Fig. S7, ESI†). This morphological transition is attributed to end-group ionization owing to deprotonation of the single carboxylic acid group located at the end of each PDMAC steric stabilizer block.^62^ Thus this remarkable thermoresponsive diblock copolymer also exhibits pH-responsive behavior.

## Conclusions

In summary, we report the one-pot PISA synthesis of a new amphiphilic PDMAC_56_–P(HBA-*stat*-DAAM)_264_ diblock copolymer that can form spheres, worms or vesicles rapidly and reversibly in aqueous solution at copolymer concentrations as low as 0.10% w/w. In addition, a vesicle-to-lamellae transition also occurs on heating to around 70 °C, but the complementary lamellae-to-vesicle transition suffers from significant hysteresis on cooling. This new system is expected to provide a suitable model for studying the kinetics and mechanism(s) of block copolymer self-assembly in solution, as well as offering a highly convenient model system for theoretical studies of thermoresponsive block copolymer nano-objects.^63^ In addition, the excellent thermoreversibility exhibited by this new system is expected to facilitate the *in situ* loading of vesicles with either nanoparticles or globular proteins/enzymes, with subsequent release of such payloads being feasible simply by lowering the solution temperature to induce a vesicle-to-worm (or vesicle-to-sphere) morphological transition.

## Conflicts of interest

There are no conflicts to declare.

## Supplementary Material

Supplementary information
