# Supplementary material for: Unique aqueous self-assembly behavior of a thermoresponsive diblock copolymer
Source: Chem Sci. 2019 Nov 12;11(2):396–402. doi: 10.1039/c9sc04197d (PMC7021201; doi:10.1039/c9sc04197d)
Supplement: Supplementary file 1 [file SC-011-C9SC04197D-s001.pdf]

Electronic Supplementary Material (ESI) for Chemical Science.
This journal is © The Royal Society of Chemistry 2019

Supporting Information for:

Unique Aqueous Self-assembly Behavior of a Thermoresponsive Diblock Copolymer

Sarah J. Byard,[1] Cate T. O'Brien,[1] Matthew J. Derry,[1] Mark Williams,[1] Oleksandr O. Mykhaylyk[1], Adam Blanazs[2] and Steven P. Armes[1]*

[1] Dainton Building, Department of Chemistry, University of Sheffield, Brook Hill, Sheffield, South Yorkshire, S3 7HF, UK.

[2] BASF SE, GMV/P-B001, 67056 Ludwigshafen, Germany.

Contents:

Materials and Methods 1

Polymer Characterization 3

Small Angle X-ray Scattering (SAXS) models 4

Supporting Figures 7

Figure S1. Assigned ^{1}H NMR spectra 7

Figure S2. Calibration plot for determining the mean DP of the PDMAC precursor 8

Figure S3. Temperature-dependent rheological data 9

Figure S4. Shear-induced polarized light imaging (SIPLI) data....................10

Figure S5. Variable temperature ^{1}H NMR spectra 11

Figure S6. Apparent z-average diameter as a function of temperature as determined by DLS 12

Figure S7. (a) Apparent z-average diameter as a function of pH as determined by DLS 13

References 13

Materials and Methods

N,N-Dimethylacrylamide (DMAC), L-ascorbic acid (AA), potassium persulfate (KPS), pyridine and 2-(dodecylthiocarbonothioylthio)-2-methylpropionic acid (DDMAT) were purchased from Sigma-Aldrich (UK) and used as received. Diacetone acrylamide (DAAM) and adipic acid dihydrazide were purchased from Alfa Aesar (UK) and used as received. Hexane, HCl and DMF were purchased from VWR chemicals (UK) and used as received. 4-Hydroxybutyl acrylate (HBA) was kindly donated by Scott Bader Ltd. (Wollaston, UK) and was purified by solvent extraction before use (see below for further details). CD_3OD was purchased from Cambridge Isotope Laboratories (UK). 1,1,2,2-Tetrachloroethane-d_2 was purchased from Goss Scientific Ltd (UK). All solvents were HPLC-grade. Deionized water was adjusted to pH 3 using HCl and used for all experiments.

Purification of 4-hydroxybutyl acrylate (HBA)

HBA (100 g) was washed with *n*-hexane (20 x 100 mL) to remove diacrylate impurities. Residual solvent was removed by rotary evaporation to yield purified HBA (70 g, 70%) as a viscous colorless fluid. ^{1}H NMR (400 MHz, CD_3OD) δ 6.37 (1H, dd), 6.15 (1H, dd), 5.87 (1H, dd), 4.18 (2H, t), 3.59 (2H, t), 1.67 (2H, m).

One-pot synthesis of PDMAC$_{56}$-P(HBA-stat-DAAM)$_{264}$ diblock copolymer nano-objects by RAFT aqueous dispersion polymerization at pH 3

Step 1 Synthesis of PDMAC precursor

KPS (9.3 mg 0.034 mmol, CTA/KPS molar ratio = 4.0) and AA (6.0 mg 0.034 mmol, CTA/AA molar ratio = 4.0) and acidified water (0.16 mL, adjusted to pH 3 with HCl) were added to a 50 mL round-bottomed flask. 2-(dodecylthiocarbonothioylthio)-2-methylpropionic acid (DDMAT) (0.050 g, 0.137 mmol) and DMAC (0.58 g, 5.89 mmol, target DP = 43) were weighed into a 14 mL vial. Both the round bottom flask and vial were purged with nitrogen gas for 15 min to remove oxygen. The DMAC/DDMAT solution was then added to the round bottom flask to produce an 80% w/w aqueous solution. The sealed flask was immersed into an oil bath set at 30°C for 30 min with continuous stirring. The sealed reaction vessel was removed from the oil bath and a further portion of degassed acidified water (2.44 g, pH 3 adjusted to pH 3 with HCl) was added. The flask was agitated for 30 minutes to produce a 20% w/w aqueous solution, which was stirred for a further 3 h at 30 °C. A 0.1 mL (3.1% of the total solution volume) aliquot was removed for ^{1}H NMR spectroscopy and GPC analysis.

^{1}H NMR spectroscopy studies indicated that > 99% DMAC conversion was achieved within 4 h, as determined by comparing the DMAC vinyl proton signals at 5.5–7.0 ppm to that of the PDMAC methyl protons signal at 2.8–3.2 ppm. DMF GPC analysis indicated a M_n of 6100 g mol^{-1} and a M_w/M_n of 1.23, respectively. A mean DP of 56 was determined for the PDMAC precursor prepared during the one-pot protocol. This DP was calculated using a calibration curve of M_p vs. DP, which was constructed for a series of nine PDMAC homopolymers, which were previously characterized by DMF GPC and UV-visible absorption spectroscopy (see Figure S2).[1] These PDMAC homopolymers were prepared using a previously published protocol.[1] The DDMAT RAFT agent efficiency during the one-pot protocol was calculated to be 77% by comparing the experimental PDMAC DP of 56 to the target DP of 43.

Step 2 Chain extension of PDMAC with HBA and DAAM

HBA (2.53 g, 17.55 mmol), DAAM (0.743 g, 4.39 mmol) and acidified water (13.1 mL, adjusted to pH 3 with HCl) were added to a 24 mL vial and the resulting comonomer solution was purged with nitrogen for 30 min. The HBA and DAAM target DPs for this RAFT aqueous dispersion copolymerization were 129 and 32 respectively ([HBA]/[DAAM] = 4) accounting for the removal of 0.1 mL PDMAC solution for analysis in step 1 and assuming 100% CTA efficiency. The degassed HBA/DAAM comonomer solution was added to the sealed round bottom flask from step 1, containing the PDMAC precursor. The sealed flask was immersed into an oil bath set at 30°C for 18 h with continuous stirring to produce a transparent free-standing gel.

^{1}H NMR spectroscopy studies indicated that more than 99% monomer conversion was achieved for both DAAM and HBA within 18 h, as determined by comparing the DAAM/HBA vinyl proton signals at 5.5–7.0 ppm to that of the PHBA oxymethylene protons signal at 3.6 ppm and the overlapping PHBA backbone proton signal and PDAAM methyl protons signal at 2.0-2.5 ppm. DMF GPC analysis indicated an M_n of 49 200 g mol^{-1} and an M_w/M_n of 1.16. An overall HBA/DAAM DP of 264 was determined by ^{1}H NMR spectroscopy by comparison of PHBA signal i, the overlapping PHBA and PDAAM signals *c* and *e*, the overlapping PDAAM and PDMAC signals *n*, *c* and *b* in Figure S2 This overall DP was higher than anticipated and could be explained by a number of factors such as RAFT agent efficiency, PDMAC blocking efficiency, small fractions of dead chain ends etc.

Post-polymerization cross-linking Using ADH

The protocol used for covalent cross-linking the $PDMAC_{56}$–P(HBA-*stat*-DAAM)$_{264}$ nano-objects was as follows. The as-synthesized $PDMAC_{56}$–P(HBA-*stat*-DAAM)$_{264}$ 20% w/w nanoparticle dispersion (0.25 g) was added to a 14 mL vial and diluted to 5% w/w solids using acidified water (distilled water adjusted to pH 3 with HCl). The 5% w/w dispersion was equilibrated at the desired temperature for 24 h. Adipic acid dihydrazide (ADH) (11.3 mg, 0.065 mmol; DAAM/ADH molar ratio = 1.0) was added to the vial, using hand-shaking to ensure uniform mixing. Cross-linking was conducted at the desired temperature for 24 h.

Polymer Characterization

^{1}H NMR spectroscopy

^{1}H NMR spectra were recorded in CD_3OD using a 400 MHz Bruker Avance III HD 400 spectrometer with 16 scans being averaged per spectrum.

Variable Temperature ^{1}H NMR spectroscopy

^{1}H NMR spectra were recorded using a 500 MHz Bruker Avance-500 spectrometer. A outer tube contained the as-synthesized 20% w/w aqueous dispersion of PDMAC-P(HBA-*stat*-DAAM) nano-objects and an inner capillary tube contained the pyridine standard in $C_2D_2Cl_4$. Spectra were recorded from 5°C to 70°C at 5°C intervals with an equilibrium time of 10 min at each temperature.

Gel permeation chromatography (GPC)

Copolymer molecular weight distributions were assessed using DMF GPC. The set-up comprised a PL guard column and two Agilent PL gel 5 μm Mixed-C columns connected in series to an Agilent 1260 Infinity GPC system equipped with both refractive index and UV-visible detectors operating at 60°C. The GPC eluent was HPLC-grade DMF containing 10 mM LiBr and the flow rate was 1.0 mL min^{-1}. Calibration was achieved using a series of ten near-monodisperse poly(methyl methacrylate) standards (ranging in M_p from 625 to 618 000 g mol^{-1}). Chromatograms were analyzed using Agilent GPC/SEC software.

Variable Temperature Dynamic Light Scattering (DLS)

The 'sphere-equivalent' intensity-average diameter of diblock copolymer nano-objects was determined using the Stokes-Einstein equation, which assumes perfectly monodisperse, non-interacting spheres. All measurements were made using a Malvern Zetasizer NanoZS instrument. 1.0 mL of a dilute (0.10% w/w) aqueous copolymer dispersion at pH 3 (diluted using distilled water adjusted to pH 3 using HCl) was placed in a glass cuvette and heated from 1°C to 50°C, followed by cooling from 50°C to 1°C at 5°C intervals with 10 min being allowed for thermal equilibration at each temperature. The intensity-average hydrodynamic diameter was determined at each temperature by averaging data over three consecutive runs.

Aqueous Electrophoresis

Zeta potential measurements were performed using a Malvern Zetasizer Nano ZS instrument on 0.1% w/w aqueous copolymer dispersions at 25°C in the presence of 1 mM KCl. The initial copolymer dispersion was acidic (pH 3) with the solution pH being adjusted by addition of dilute NaOH to pH 8 followed by adjustment with dilute HCl to pH 3, with 10 min being allowed for equilibrium at each pH. Zeta potentials were calculated from the Henry equation using the Smoluchowski approximation. Hydrodynamic DLS diameters were also recorded during these pH experiments. All data were averaged over three consecutive runs.

UV−Visible Absorption Spectroscopy

UV−visible absorption spectra were recorded between 200 and 800 nm using a PC-controlled UV-1800 spectrophotometer at 25°C using a 1.0 cm path length quartz cell. The DPs for a series of nine PDMAC homopolymers were determined using a molar extinction coefficient of 16300 ± 160 mol^{-1} dm^3 cm^{-1} for DDMAT as reported in a previous publication.[1]

Transmission Electron Microscopy (TEM)

Copper/palladium TEM grids (Agar Scientific, UK) were coated in-house to yield a thin film of amorphous carbon. The coated grids were then subjected to a glow discharge for 30 s. An individual 10.0 μL droplet for each 0.10% w/w aqueous copolymer dispersion was placed on a freshly-treated TEM grid for 1 min and then carefully blotted with filter paper to remove excess solution. To ensure sufficient electron contrast, uranyl formate (9.0 μL of a 0.75% w/w solution) was absorbed onto the sample-loaded grid for 30 s and then carefully blotted to remove

excess stain. Each grid was then dried using a vacuum hose. Imaging was performed using a FEI Tecnai Spirit 2 microscope operating at 80 kV and fitted with an Orius SC1000B camera. Particle sizes were calculated using image J. An average size was calculated by analyzing 100 particles.

Rheology

An AR-G2 rheometer equipped with a variable-temperature Peltier plate and a 40 ml 2° aluminium cone was used for all experiments. Temperature sweeps were conducted using a constant percentage strain of 1.0% and a constant angular frequency of 1.0 rad s^{-1}. Before the temperature sweep, the aqueous $PDMAC_{56}$–P(HBA-*stat*-DAAM)$_{264}$ copolymer dispersion was equilibrated at 1°C for 15 min. A ramp rate of 1.0 °C min^{-1} was used for all experiments.

Shear-induced polarized light imaging (SIPLI) experiments

The instrument design and general experimental set-up has been previously reported by Mykhaylyk and co-workers (see ref. 57 in the main manuscript). SIPLI experiments were conducted on a 20% w/w aqueous dispersion of $PDMAC_{56}$–P(HBA-*stat*-DAAM)$_{264}$ nano-objects at an applied shear rate of 1.0 s^{-1} during a temperature ramp experiment conducted at a heating/cooling rate of 1.0 °C min^{-1}.

Small Angle X-Ray scattering (SAXS)

SAXS patterns were recorded at a synchrotron source (ESRF, station ID02, Grenoble, France) using monochromatic X-ray radiation (X-ray wavelength λ = 0.0995 nm, scattering vector q ranging from 0.0015 to 0.15 $Å^{-1}$, where $q = 4\pi \sin \theta/\lambda$ and θ is one-half of the scattering angle) and a Ravonix MX-170HS CCD detector. A glass capillary of 2 mm diameter was used as a sample holder and the temperature was controlled using a heating/cooling capillary holding stage (Linkam Scientific Instruments Ltd., Tadworth, UK). Measurements were conducted on a 1.0% w/w aqueous dispersion of $PDMAC_{56}$–P(HBA-*stat*-DAAM)$_{264}$ particles in pH 3 water (adjusted using HCl). Scattering data was recorded from 5°C to 70°C at a heating rate of 1°C min^{-1}. Scattering data were reduced (normalization and integration) using standard routines available at the beamline and were further analyzed using Irena SAS macros for Igor Pro.

Small Angle X-ray Scattering (SAXS) models

In general, the intensity of X-rays scattered by a dispersion of nano-objects [usually represented by the scattering cross section per unit sample volume, $\frac{d\Sigma}{d\Omega}(q)$] can be expressed as:

$$\frac{d\Sigma}{d\Omega}(q) = NS(q)\int_0^\infty \dots \int_0^\infty F(q,r_1,\dots,r_k)^2 \Psi(r_1,\dots,r_k)dr_1\dots dr_k \qquad \text{S1}$$

where $F(q,r_1,\dots,r_k)$ is the form factor, $r_1,\dots,r_k$ is a set of k parameters describing the structural morphology, $\Psi(r_1,\dots,r_k)$ is the distribution function, $S(q)$ is the structure factor and N is the nano-object number density per unit volume expressed as:

$$N = \frac{\varphi}{\int_0^\infty \dots \int_0^\infty V(r_1,\dots,r_k)\Psi(r_1,\dots,r_k)dr_1\dots dr_k} \qquad \text{S2}$$

where $V(r_1,\dots,r_k)$ is volume of the nano-object and φ is their volume fraction in the dispersion. Since all SAXS data were collected on dilute nanoparticle dispersions (1.0% w/w), it can be assumed that there are no interparticle interactions and thus $S(q) = 1$.

Spherical micelle model

The spherical micelle form factor for Equation S1 is given by:[2]

$$F_{s_{mic}}(q) = N_s^2\beta_s^2A_s^2(q,R_s) + N_s\beta_c^2F_c(q,R_g) + N_s(N_s - 1)\beta_c^2A_c^2(q) + 2N_s^2\beta_s\beta_cA_s(q,R_s)A_c(q)$$
S3

where R_s is the core radius of the spherical micelle, R_g is the radius of gyration of the PDMAC corona block. The core block and the corona block X-ray scattering length contrast is given by $\beta_s = V_s(\xi_s - \xi_{sol})$ and $\beta_c = V_c(\xi_c - \xi_{sol})$, respectively. Here ξ_s, ξ_c and ξ_{sol} are the X-ray scattering length densities of the core block ($\xi_{PHBA-PDAAM} = 10.56 \times 10^{10}$ cm^{-2}), the corona block ($\xi_{PDMAC} = 10.72 \times 10^{10}$ cm^{-2}) and the solvent ($\xi_{sol} = 9.42 \times 10^{10}$ cm^{-2}), respectively. V_s and V_c are volumes of the core block ($V_{PHBA-PDAAM}$) and the corona block (V_{PDMAC}), respectively. The volumes were obtained from $V = \frac{M_{n,pol}}{N_A\rho}$ using the densities of PHBA ($\rho_{PHBD} = 1.16$ g cm^{-3}), PDAAM ($\rho_{PDAAM} = 1.13$ g cm^{-3}) and PDMAC ($\rho_{PDMAC} = 1.16$ g cm^{-3}), where $M_{n,pol}$ corresponds to the number-average molecular weight of the block determined by ^{1}H NMR spectroscopy. The sphere form factor amplitude is used for the amplitude of the core self-term:

$$A_c(q,R_s) = \Phi(qR_s)exp^{\left(-\frac{q^2\sigma^2}{2}\right)}$$
S4

where $\Phi(qR_s) = \frac{3[\sin(qR_s) - qR_s\cos(qR_s)]}{(qR_s)^3}$. A sigmoidal interface between the two blocks was assumed for the spherical micelle form factor [Equation S3]. This is described by the exponent term with a width σ accounting for a decaying scattering length density at the micellar interface. This σ value was fixed at 2.5 during fitting. The form factor amplitude of the spherical micelle corona is:

$$A_c(q) = \frac{\int_{R_s}^{R_s+2s} \mu_c(r)\frac{\sin(qr)}{qr}r^2dr}{\int_{R_s}^{R_s+2s} \mu_c(r)r^2dr}exp^{\left(-\frac{q^2\sigma^2}{2}\right)}$$
S5

The radial profile, $\mu_c(r)$, can be expressed by a linear combination of two cubic b splines, with two fitting parameters s and a corresponding to the width of the profile and the weight coefficient, respectively. This information can be found elsewhere,[34] as can the approximate integrated form of Equation S5. The self-correlation term for the corona block is given by the Debye function:

$$F_c(q,R_g) = \frac{2\left[e^{\left(-q^2R_g^2\right)} - 1 + q^2R_g^2\right]}{q^4R_g^4}$$
S6

where R_g is the radius of gyration of the PDMAC coronal block. The aggregation number of the spherical micelle is:

$$N_s = (1 - x_{sol})\frac{\frac{4}{3}\pi R_s^3}{V_s}$$
S7

where x_{sol} is the volume fraction of solvent in the PHBA-PDAAM micelle core. A polydispersity for one parameter (R_s) is assumed for the micelle model which is described by a Gaussian distribution. Thus, the polydispersity function in Equation S1 can be represented as:

$$\Psi(r_1) = \frac{1}{\sqrt{2\pi\sigma_{R_s}^2}} exp^{\left(-\frac{(r_1 - R_s)^2}{2\sigma_{R_s}^2}\right)} \qquad S8$$

where σ_{R_s} is the standard deviation for R_s. In accordance with Equation S2, the number density per unit volume for the micelle model is expressed as:

$$N = \frac{\varphi}{\int_0^\infty V(r_1)\Psi(r_1)dr_1} \qquad S9$$

where φ is the total volume fraction of copolymer in the spherical micelles and $V(r_1)$ is the total *volume* of copolymer in a spherical micelle $[V(r_1) = (V_s + V_c)N_s(r_1)]$.

The experimental R_g obtained from this fitting for the corona PDMAC block (1.7 nm) is also physically reasonable, since it is close to the estimated parameter. Assuming that the contour length of a PDMAC monomer is 0.255 nm (two C-C bonds in all-trans conformation), the total contour length of a $PDMAC_{56}$ block, $L_{PDMAC_{56}}$ = 56 × 0.255 nm = 14.28 nm. Given a mean Kuhn length of 1.53 nm [based on the known literature value for PMMA[5]] an estimated unperturbed radius of gyration, $R_g = (14.28 \times 1.13/6)^{0.5}$, or 1.91 nm is determined.

Worm-like micelle model
The worm-like micelle form factor in Equation S1 is expressed as:[2]

$$F_{w_{mic}}(q) = N_w^2\beta_s^2F_w(q) + N_w\beta_c^2F_c(q,R_g) + N_w(N_w - 1)\beta_c^2S_{cc}(q) + 2N_w^2\beta_s\beta_cS_{sc}(q) \qquad S10$$

where all the parameters are the same as in the spherical micelles model (Equation S3) unless stated otherwise. The self-correlation time for the worm-like micelle core or radius R_w is:

$$F_w(q) = F_{worm}(q,L_w,b_w)A_{csworm}^2(q,R_w) \qquad S11$$

Where

$$A_{csworm}^2(q,R_w) = \left[2\frac{J_1(qR_w)}{qR_w}\right]^2 \qquad S12$$

and J_1 is the first-order Bessel function of the first kind, and a form factor $F_{worm}(q,L_w,b_w)$ for self-avoiding semi-flexible chains represent the worm-like micelle, where b_w is the worm Kuhn length and L_w is the mean worm contour length. A complete expression for the chain form factor can be found elsewhere.[6] The self-correlation term for the corona block is given by the Debye function shown in Equation S6.
The mean aggregation number of the worm-like micelle is given by:

$$N_w = (1 - x_{sol})\frac{\pi R_w^2L_w}{V_s} \qquad S13$$

where x_{sol} is the volume fraction of solvent within the worm-like micelle core. Possible semi-spherical caps at the ends of each worm are not considered in this form factor. Again, the obtained R_g of the $PDMAC_{56}$ coronal block of 1.9 nm is comparable to the estimated value of 1.91 nm.

Vesicle model
The vesicle form factor in Equation S1 is expressed as:[7]

$$F_{ves}(q) = N_v^2\beta_s^2A_m^2(q) + N_v\beta_c^2F_c(q,R_g) + N_v(N_v - 1)\beta_c^2A_{vc}^2(q) + 2N_v^2\beta_s\beta_cA_m(q)A_{vc}(q) \quad \text{S14}$$

where all the parameters are the same as in the spherical micelles model (Equation S3) unless stated otherwise. The amplitude of the membrane self-term is:

$$A_m(q) = \frac{V_{out}\varphi(qR_{out}) - V_{in}\varphi(qR_{in})}{V_{out} - V_{in}}exp\left(-\frac{q^2\sigma_{in}^2}{2}\right) \quad \text{S15}$$

where $R_{in} = R_m - \frac{1}{2}T_m$ is the inner radius of the membrane, $R_{out} = R_m + \frac{1}{2}T_m$ is the outer radius of the membrane (R_m is the radius from the centre of the vesicle to the centre of the membrane), $V_{in} = \frac{4}{3}\pi R_{in}^3$, and $V_{out} = \frac{4}{3}\pi R_{out}^3$. It should be noted that Equation S14 differs from the original work in which they were first described.[7] The exponent term in Equation S15 represents a sigmoidal interface between the blocks, with a width σ_{in} accounting for a decaying scattering length density at the membrane surface. The value of σ_{in} was fixed at 2.5. The mean vesicle aggregation number, N_v, is given by:

$$N_v = (1 - x_{sol})\frac{V_{out} - V_{in}}{V_s} \quad \text{S16}$$

where x_{sol} is the solvent (i.e. water) volume fraction within the vesicle membrane. Assuming that there is no penetration of the hydrophilic coronal blocks into the hydrophobic membrane, the amplitude of the vesicle corona self-term is expressed as:

$$A_{vc}(q) = \Psi(qR_g)\frac{1}{2}\left[\frac{sin[q(R_{out} + R_g)]}{q(R_{out} + R_g)} + \frac{sin[q(R_{in} - R_g)]}{q(R_{in} - R_g)}\right] \quad \text{S17}$$

where the term outside the square brackets is the factor amplitude of the corona block polymer chain such that:

$$\Psi(qR_g) = \frac{1 - exp(-qR_g)}{(qR_g)^2} \quad \text{S18}$$

Again, the obtained R_g of the $PDMAC_{56}$ coronal block of 1.7 nm is comparable to the estimated value of 1.91 nm. It was assumed for the vesicle model that two parameters are polydisperse: the the radius from the centre of the vesicle to the centre of the membrane and the membrane thickness (R_m and T_m, respectively). They are considered to have a Gaussian distribution and, therefore, the polydispersity function in Equation S1 can be expressed as:

$$\Psi(r_1r_2) = \frac{1}{\sqrt{2\pi\sigma_{R_m}^2}}exp\left(-\frac{(r_1 - R_m)^2}{2\sigma_{R_m}^2}\right)\frac{1}{\sqrt{2\pi\sigma_{T_m}^2}}exp\left(-\frac{(r_1 - T_m)^2}{2\sigma_{T_m}^2}\right) \quad \text{S19}$$

where σ_{R_m} and σ_{T_m} are the standard deviations for $R_m$and T_m, respectively. Following Equation S2 the number density per unit volume for the vesicle model is expressed as:

$$N = \frac{\varphi}{\int_0^\infty \int_0^\infty V(r_1,r_2)\Psi(r_1,r_2)dr_1dr_2} \quad \text{S20}$$

where φ is the total volume fraction of copolymer in the vesicles and $V(r_1,r_2)$ is the total *volume* of copolymers in a vesicle $[V(r_1,r_2) = (V_s + V_c)N_v(r_1,r_2)]$. Programming tools within the Irena SAS Igor Pro macros were used to implement the scattering models.[8]

Rheo-SAXS measurements

This experiment was performed using a SAXS laboratory instrument (Xeuss 2.0, Xenocs, France) equipped with a liquid gallium MetalJet X-ray source (Excillum, Sweden, X-ray wavelength λ = 1.34 Å^{-1}) and a 2D Pilatus 1M pixel detector (Dectris, Switzerland). SAXS patterns were collected over a q range of 0.0025 Å^{-1} < q < 0.15 Å^{-1} using sample-to-detector distance of 5.20 m. A CSS 450 shear cell (Linkam, Tadworth, UK) with a 0.75 mm shear gap modified with steel disks with a hole (static disk) and circularly-segmented milled slots (shearing disk) covered by Kapton™ films was used for the *in situ* rheo-SAXS measurements. A few grams of a 20% w/w aqueous dispersion of $PDMAC_{56}$–P(HBA-*stat*-DAAM)$_{264}$ diblock copolymer nano-objects (which conveniently forms a free-standing gel at ambient temperature) was loaded into the rotational parallel plate shear cell while horizontal, then the shear cell was mounted vertically in the SAXS instrument. The temperature of the shear cell was then raised from 25 °C to 63 °C at a heating rate of 10 °C min^{-1} and equilibrated for 2 min prior to applying a large-amplitude oscillatory shear at a frequency of 0.05 Hz and a strain amplitude of 2000%. SAXS patterns were collected at a rate of two frames per minute. Representative 2D SAXS patterns obtained with and without shear are shown in Figure 4b.

Supporting Figures

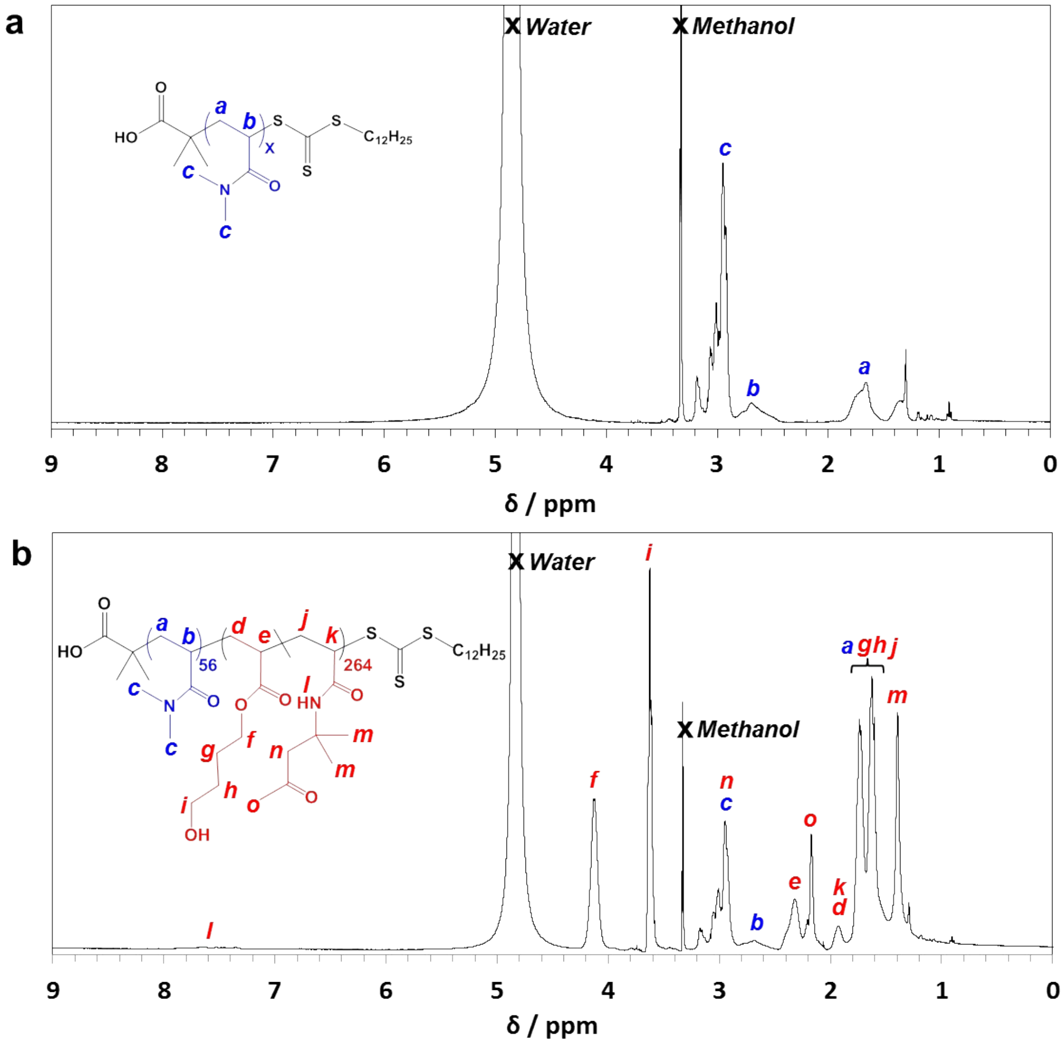

Figure S1. Assigned ^{1}H NMR spectra recorded in CD_3OD for (a) the $PDMAC_{56}$ precursor and (b) the $PDMAC_{56}$–P(HBA-*stat*-DAAM)$_{264}$ diblock copolymer.

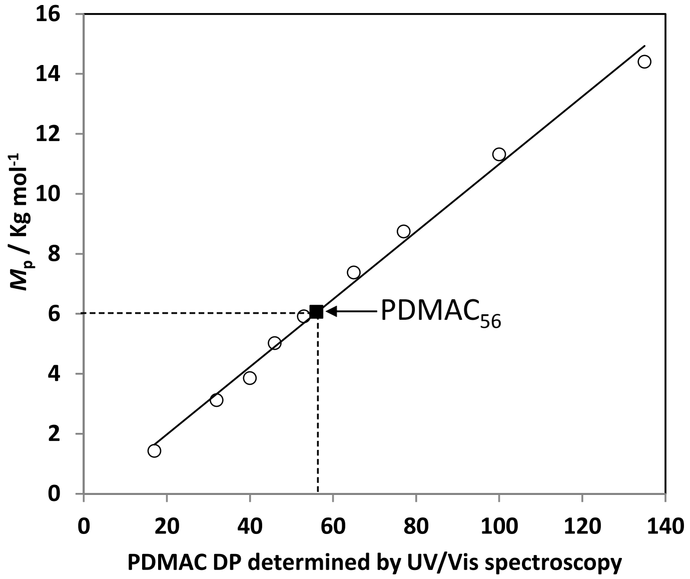

Figure S2. Calibration plot for determining the mean DP of the PDMAC precursor prepared during the one-pot protocol. M_p (determined by DMF GPC analysis using a refractive index detector) vs. PDMAC DP (determined by UV/visible absorption spectroscopy using a previously published Beer-Lambert calibration plot constructed for DDMAT[1]) for a series of PDMAC homopolymers synthesized by RAFT solution polymerization of DMAC in dioxane using DDMAT and purified by precipitation (open black circles). Black square: M_p for the $PDMAC_{56}$ precursor synthesized by RAFT solution polymerization of DMAC in pH 3 water using DDMAT at 30°C via the one-pot protocol.

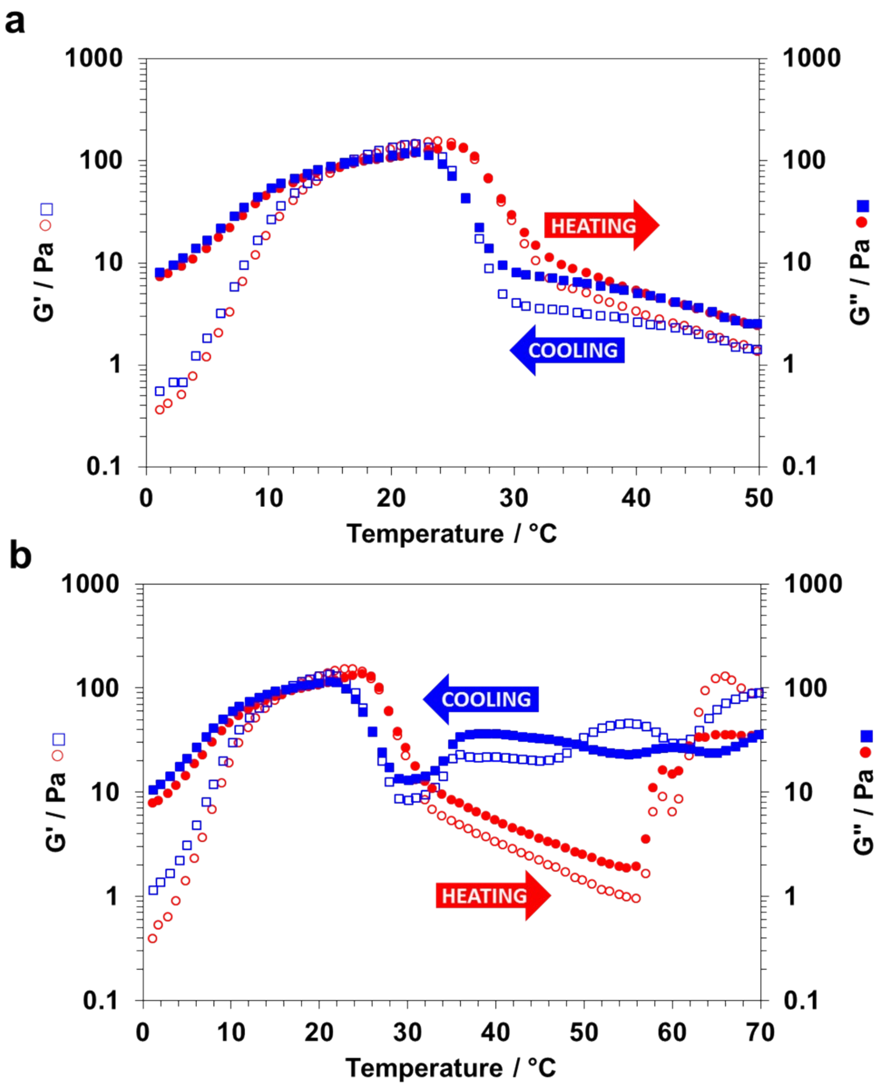

Figure S3. Temperature-dependent rheological studies for a 20% w/w dispersion of PDMAC$_{56}$-P(HBA-*stat*-DAAM)$_{264}$ aqueous nano-object dispersion at an applied strain of 1.0% and an angular frequency of 1.0 rad s$^{-1.}$ - The dispersion was equilibrated at 1°C for 15 min prior to heating. **A** G' and G" as a function of temperature for a heating-cooling cycle from 1°C to 50°C to 1°C at 1°C min^{-1}. Red open circles: G' heating, red filled cirlces: G" heating, blue open squares: G' cooling, blue filled squares: G" cooling. **B** G' and G" as a function of temperature for a heating-cooling cycle from 1°C to 70°C to 1°C at 1°C min^{-1}. Red open circles: G' heating, red filled cirlces: G" heating, blue open squares: G' cooling, blue filled squares: G" cooling.

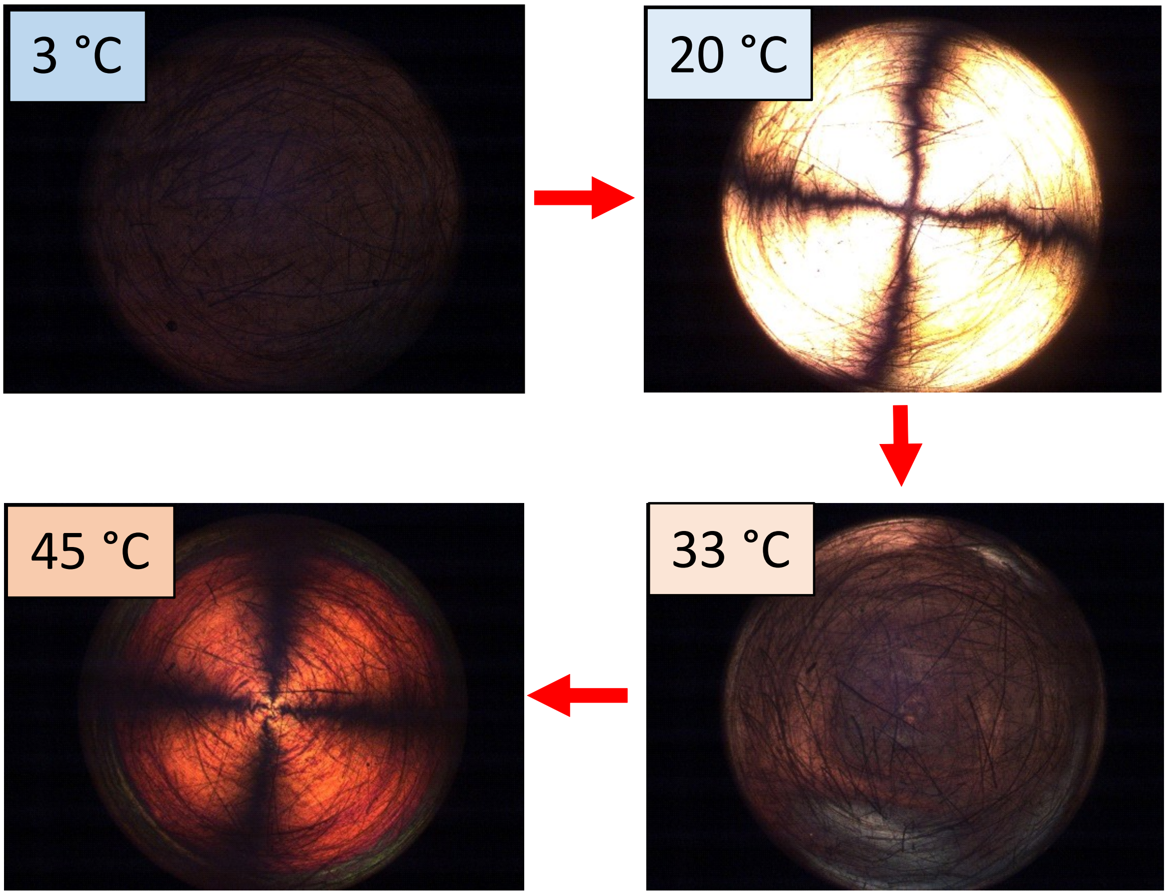

Figure S4. Shear-induced polarized light images (SIPLI) obtained for a 20% w/w aqueous dispersion of $PDMAC_{56}$-P(HBA-*stat*-DAAM)$_{264}$ at an applied shear rate of 1.0 s^{-1} during a temperature ramp experiment conducted at a heating rate of 1.0 °C min^{-1}. The initial dark image recorded at 3 ° C is consistent with the presence of isotropic spheres. In contrast, the highly anisotropic worms formed at 20 °C exhibit a characteristic Maltese cross owing to birefringence caused by their alignment in the direction of shear flow. This distinctive feature disappears at 33 °C, indicating the formation of isotropic vesicles. A second Maltese cross is observed at 45 °C, which is consistent with the presence of highly anisotropic block copolymer lamellae (see ref. 57 in the main manuscript). The associated multi-coloured rings indicate stronger birefringence than that observed at 20 °C.

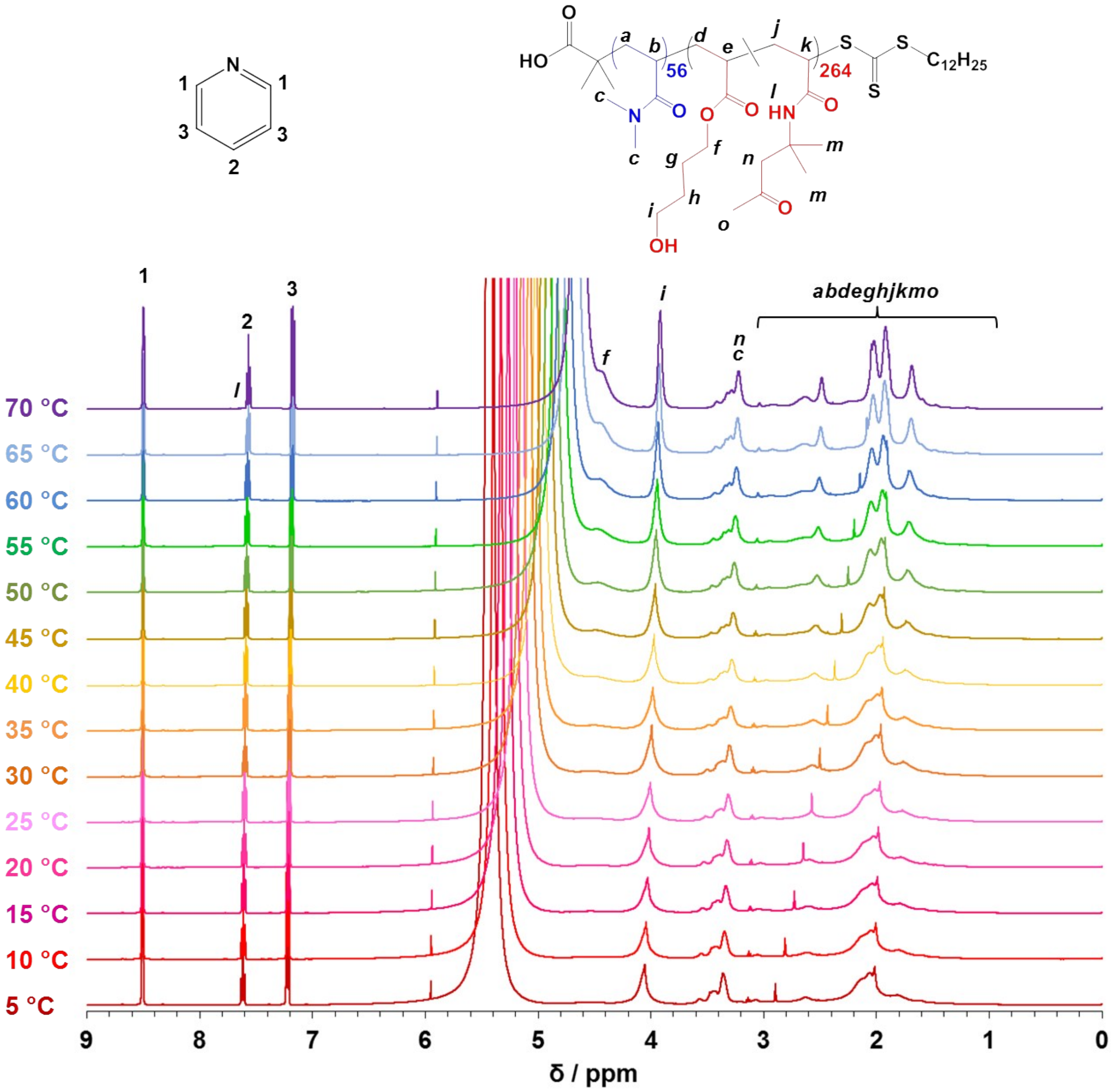

Figure S5. Variable temperature ^{1}H NMR spectroscopy study. Assigned 500 MHz ^{1}H NMR spectra recorded from 5°C to 70°C for a 20% w/w aqueous dispersion of $PDMAC_{56}$–P(HBA-*stat*-DAAM)$_{264}$ nano-objects. This dispersion was equilibrated at each temperature for 10 min to ensure thermal equilibrium. All spectra were normalized relative to proton signal **1** assigned to the external standard (pyridine).

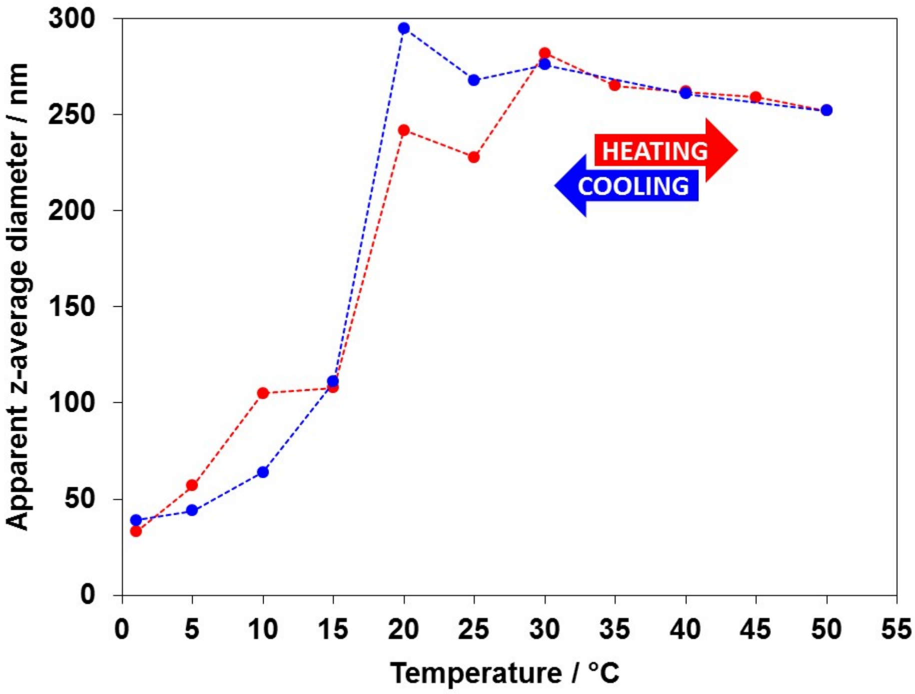

Figure S6. Apparent z-average diameter as a function of temperature as determined by DLS studies of a 0.1% w/w aqueous dispersion of $PDMAC_{56}$-P(HBA-*stat*-$DAAM)_{264}$ at pH 3. This dispersion was heated from 1°C to 50°C (red data) followed by cooling from 50°C to 1°C (blue data). The dispersion was equilibrated at each temperature for 10 min prior to DLS measurements to ensure thermal equilibration.

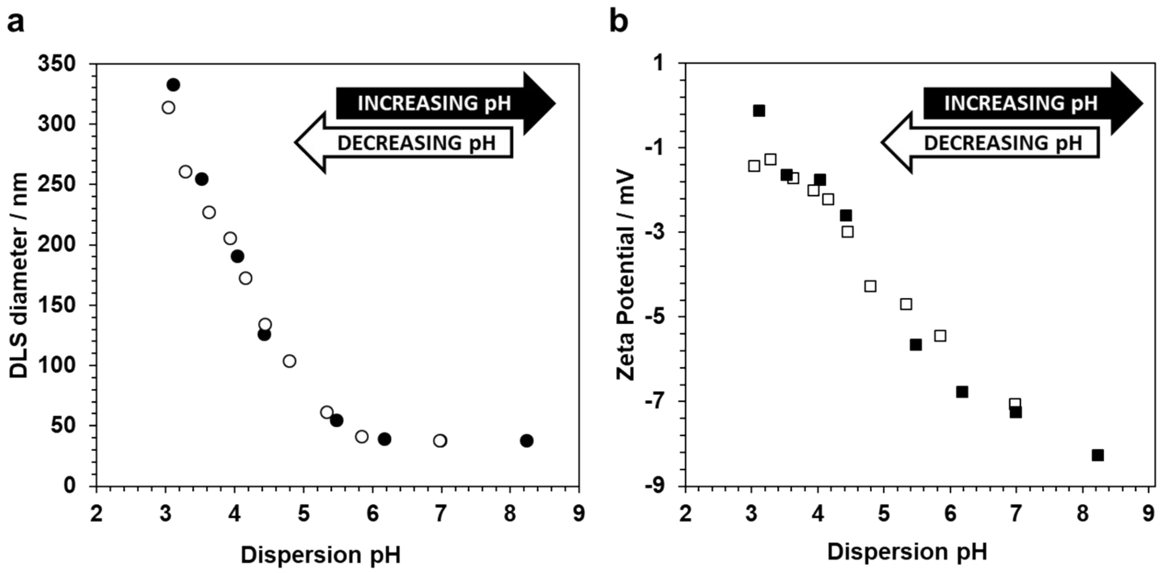

Figure S7. (a) Apparent z-average diameter as a function of pH as determined by DLS studies a 0.10% w/w aqueous dispersion of $PDMAC_{56}$-P(HBA-*stat*-DAAM)$_{264}$ nano-objects on increasing the $PDMAC_{56}$-P(HBA-*stat*-DAAM)$_{264}$ dispersion pH from pH 3 to pH 7 (filled circles) followed by returning the dispersion pH to pH 3 (empty circles) at 25 °C. The dispersion was equilibrated at each pH for 10 min. (b) Zeta potentials recorded for a 0.10% w/w aqueous dispersion of $PDMAC_{56}$-P(HBA-*stat*-DAAM)$_{264}$ nano-objects on increasing the $PDMAC_{56}$-P(HBA-*stat*-DAAM)$_{264}$ dispersion pH from pH 3 to pH 7 (filled squares) followed by returning the dispersion pH to pH 3 (empty squares). The dispersion was equilibrated at each pH for 10 min.

References

(1) Byard, S. J.; Williams, M.; Mckenzie, B. E.; Blanazs, A.; Armes, S. P. Preparation and Cross-Linking of All-Acrylamide Diblock Copolymer Nano-Objects via Polymerization-Induced Self-Assembly in Aqueous Solution. *Macromolecules* **2017**, *50*, 1482–1493.

(2) Pedersen, J. S. Form Factors of Block Copolymer Micelles with Spherical, Ellipsoidal and Cylindrical Cores. *J. Appl. Crystallogr.* **2000**, *33*, 637–640.

(3) Pedersen, J. S.; Gerstenberg, M. C. The Structure of P85 Pluronic Block Copolymer Micelles Determined by Small-Angle Neutron Scattering. *Colloids Surfaces A Physicochem. Eng. Asp.* **2003**, *213*, 175–187.

(4) Pedersen, J. S.; Svaneborg, C.; Almdal, K.; Hamley, I. W.; Young, R. N. A Small-Angle Neutron and x-Ray Contrast Variation Scattering Study of the Structure of Block Copolymer Micelles: Corona Shape and Excluded Volume Interactions. *Macromolecules* **2003**, *36*, 416–433.

(5) Fetters, L. J.; Lohsey, D. J.; Colby, R. H. *Physical Properties of Polymers Handbook*, Second.; Mark, J. E., Ed.; Springer: New York, 2007.

(6) Pedersen, J. S.; Schurtenberger, P. Scattering Functions of Semiflexible Polymers with and without Excluded Volume Effects. *Macromolecules* **1996**, *29*, 7602–7612.

(7) Bang, J.; Jain, S.; Li, Z.; Lodge, T. P.; Pedersen, J. S.; Kesselman, E.; Talmon, Y. Sphere, Cylinder, and Vesicle Nanoaggregates in Poly(Styrene-b-Isoprene) Diblock Copolymer Solutions. *Macromolecules* **2006**, *39*, 1199–1208.

(8) Ilavsky, J.; Jemian, P. R. Irena : Tool Suite for Modeling and Analysis of Small-Angle Scattering. *J. Appl. Crystallogr.* **2009**, *42*, 347–353.
